# Implementation of AI-Driven Diagnostic Tools to Improve Access and Efficiency in Rural Healthcare: An Umbrella Review

**DOI:** 10.7759/cureus.101326

**Published:** 2026-01-12

**Authors:** Hillary C Ugwu, Okiemute R Obodo, Chidiogo N Okafor, Gift Ojukwu, Edediong Ekarika, Toluwalope F Ejiyooye, Okelue E Okobi, Seun S Odusanmi, Ugochukwu N Ugwu

**Affiliations:** 1 Intensive Care Unit, Prince Mutaib bin Abdulaziz Hospital, Sakaka, SAU; 2 Medicine Department, Windsor University School of Medicine, Cayon, KNA; 3 Medicine Department, Enugu State University of Science and Technology College of Medicine, Enugu, NGA; 4 General Practice Department, Leeds Teaching Hospitals, Leeds, GBR; 5 Public Health Department, Emory University Rollins School of Public Health, Atlanta, USA; 6 Medicine Department, All Saints University School of Medicine, Roseau, DMA; 7 Family Medicine Department, Brooke Army Medical Center, San Antonio, USA; 8 Family Medicine Department, Larkin Community Hospital Palm Springs Campus, Hialeah, USA; 9 Clinical Hematology Department, University Hospitals of Leicester, Leicester, GBR; 10 Public Health Sciences Department, Teesside University, Middlesbrough, GBR

**Keywords:** ai-driven diagnostic, artificial intelligence, diagnostic tools, healthcare infrastructure, machine learning, rural healthcare, telehealth

## Abstract

Rural and underserved communities continue to face barriers to timely and accurate healthcare due to shortages of specialists, limited diagnostic infrastructure, and geographic isolation. Artificial intelligence (AI)-driven diagnostic tools, including machine learning (ML) algorithms, telehealth platforms, and clinical decision support systems, have the potential to address these challenges. A systematic review was conducted in accordance with Preferred Reporting Items for Systematic Reviews and Meta-Analyses (PRISMA) guidelines. PubMed, Scopus, Web of Science, and Embase were searched for studies published between January 2010 and April 2025 that evaluated AI-based diagnostic interventions in rural or low-resource settings. Findings were synthesized thematically to assess diagnostic performance, healthcare access, efficiency, and implementation factors. Twenty-six studies met the inclusion criteria, including observational studies, implementation case reports, and systematic reviews. Overall, AI tools were associated with improved diagnostic accuracy, reduced turnaround times, and enhanced access to services through mobile and telehealth applications. Commonly reported barriers included limited digital infrastructure, gaps in provider training, data privacy concerns, and regulatory uncertainty, while enabling factors included community trust, integration with existing health systems, and supportive policy environments. AI-driven diagnostics therefore show considerable promise for reducing inequities in rural healthcare, although successful implementation will require context-specific strategies, sustained infrastructure investment, and strong ethical and regulatory oversight.

## Introduction and background

Access to quality healthcare in rural and underserved communities is one of the most long-standing challenges in world health [1]. Rural communities may face numerous challenges to adequate and timely diagnosis, irrespective of the intention to improve the infrastructure and produce more resources [2]. These factors are few numbers of specialized healthcare providers, far travel distances to health facilities, and diagnosis equipment shortages [3]. The presence of such barriers has led to delayed diagnosis, the inadequate diagnosis of chronic conditions, and worse health outcomes relative to the populations in urban areas [4,5]. Increasing the level of access and efficiency of diagnostics, thus, is an important step toward the mitigation of health inequities and improving the capacity of healthcare delivery in rural areas [6].

Artificial intelligence (AI) calls for a new era of technologies in healthcare, providing solutions in disease detection, prognosis, and clinical decision support [7]. Machine learning (ML), image recognition, and natural language processing are among AI-powered diagnostic tools that have shown potential to enhance the accuracy, efficiency, and reach of healthcare diagnoses [8]. Training on huge data sets of clinical information and imaging shows that AI systems can help healthcare workers with diagnosing diseases such as tuberculosis (TB), diabetes, cancers, and cardiovascular diseases at a faster rate and with more accuracy than general methods [9]. Notably, they are also applicable in resource-constrained environments using mobile solutions and cloud-based systems, where their applicability in rural healthcare is quite appropriate [10].

Although it looks promising, the adoption of AI diagnostic tools in rural healthcare is a complicated matter and dependent on various factors [11]. On the one hand, AI tools may contribute to a reduction in the reliance on rare specialists and provide community health workers with more decision support, which will enhance access to healthcare [12]. Conversely, issues such as poor digital infrastructure, the incompetent training of healthcare providers, data privacy doubts, and cultural acceptability can restrict their effective implementation [13]. Additionally, the ethical and regulatory AI in healthcare aspects is immature and can create an obstacle to inclusion in rural health systems [14].

Although the number of studies devoted to discovering AI applications in healthcare is growing, no systematic study of evidence targeting specifically the rural context exists [15]. Available literature can be considered incomplete because they focus on either the technical aspect of performance or the implementation issues but do not provide an integrated synthesis of the effects of AI-based diagnostic tools on access and efficiency in these medically underserved populations [11]. This gap needs to be addressed to provide policymakers, health professionals, and technology developers with the knowledge on how to successfully implement it.

This systematic review will, therefore, attempt to review and combine available evidence on the application of AI-based diagnostic systems in rural medical practice. The main objective of the study is to identify how these tools contribute to healthcare access and efficiency and what impediments and enablers affect their adoption. Synthesizing the findings of various projects, this review is expected to assist practitioners in developing evidence-based recommendations on the improvement of healthcare delivery in rural communities via AI innovations.

## Review

Eligibility criteria and search strategies

**Figure 1:**
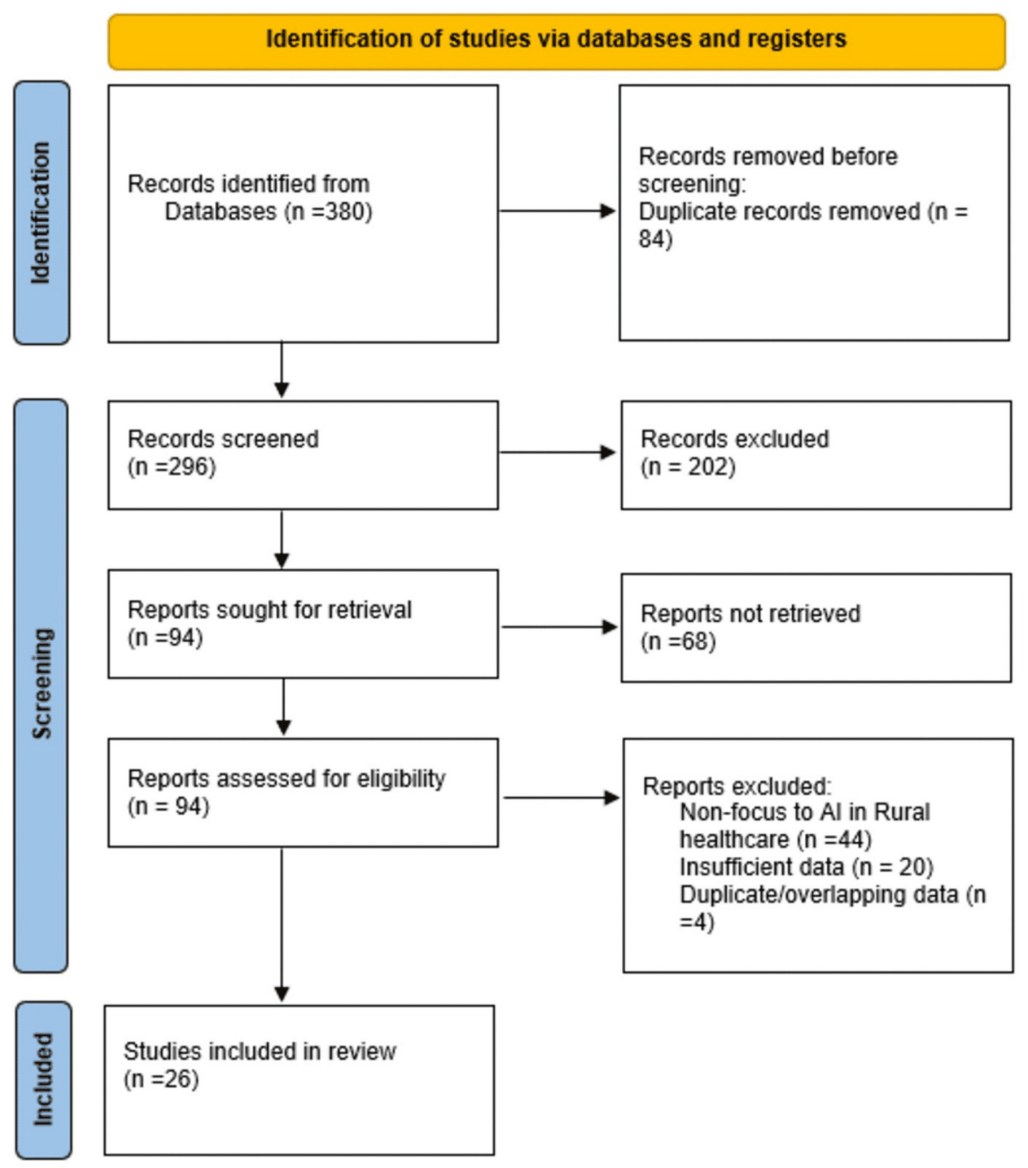
PRISMA flow diagram indicating the study selection and inclusion process Source: [16] PRISMA, Preferred Reporting Items for Systematic Reviews and Meta-Analyses; AI, artificial intelligence

To be rigorous and transparent, this systematic review was done in accordance with the Preferred Reporting Items for Systematic Reviews and Meta-Analyses (PRISMA) 2020 guidelines, as shown in Figure 1 [16]. This review was designed as an umbrella-style synthesis of review-level evidence, focusing on systematic, scoping, and policy reviews examining AI-enabled diagnostic tools in rural and underserved healthcare settings. The population, intervention, comparison, and outcome (PICO) model, as applied in previous systematic reviews, was used to frame the research question: population (rural populations and healthcare workers in low-resource or underserved settings), intervention (AI-driven diagnostic tools, such as machine learning, deep learning {DL}, and digital decision support systems), comparison (not required, as review asked about the implementation and outcomes without necessarily comparing them to traditional approaches), and outcomes (improved access to medical services, increased efficiency, diagnostic accuracy, timeliness, and the presence of barriers/facilitators to implementation) [1].

Only studies published between January 2010 and April 2025 were considered, as during this time, an interface between AI and healthcare started to develop rapidly. Eligible articles were identified through a comprehensive search across PubMed, Scopus, Web of Science, and Embase. They included peer-reviewed publications published in English only. The search strategy encompassed both MeSH terms and keywords, bound together by a combination of Boolean operators, as shown in Table 1. Reference lists of included articles and related systematic reviews were also screened for additional studies.

**Table 1:** Overview of search strategy

Category	Details
Databases searched	PubMed, Scopus, Embase, and Web of Science
Time frame	January 2010 to April 2025
Language	English only
Search terms	#1 AND #2 AND #3
#1 (population)	“Rural health” OR “rural healthcare” OR “low-resource settings” OR “underserved populations”
#2 (intervention)	“Artificial intelligence” OR “AI tools” OR “machine learning” OR “digital diagnostics” OR “clinical decision support” OR “telehealth diagnostics”
#3 (outcome)	“Access to healthcare” OR “healthcare efficiency” OR “diagnostic accuracy” OR “implementation barriers” OR “implementation facilitators”

Inclusion criteria

Studies were eligible for inclusion if they examined AI-enabled diagnostic technologies or digital decision support systems relevant to rural, remote, underserved, or low-resource healthcare settings. Eligible study designs included systematic reviews, meta-analyses, scoping reviews, qualitative review syntheses, policy reviews, and mixed-methods reviews that synthesized evidence on diagnostic access, implementation barriers, facilitators, ethical considerations, or health system impacts.

Exclusion criteria

Publications were excluded if they were editorials, letters to the editor, commentaries, conference abstracts, dissertations, study protocols without results, or non-peer-reviewed articles. Studies published in languages other than English were excluded. Additionally, articles that addressed AI in healthcare without a diagnostic focus or without relevance to rural, underserved, or low-resource contexts were not included.

Screening process

All found articles were imported into EndNote (Clarivate, London, UK) reference manager to remove duplicates. Titles and abstracts were screened by two reviewers independently to determine eligibility. Potentially relevant articles were then screened by full text. Any disagreements were settled by discussion, with a tie-breaking third reviewer resolving minor conflicts that they could not come to an agreement over.

Quality assessment

In assessing the methodological quality, suitable instruments were applied based on the study design. The Revised Assessment of Multiple Systematic Reviews (R-AMSTAR) tool was applied to assess the selected articles [17].

Data extraction

A standardized form was created to identify the following information: study title, author(s), year of publication, country, study design, population characteristics, the type of AI-driven diagnostic tool, healthcare setting, outcomes measured (access, efficiency, accuracy, barriers, and facilitators), and key findings. Data extraction was performed by two reviewers independently and cross-checked for accuracy.

Data analysis

The extracted data were synthesized thematically. Studies were grouped into categories: (1) AI effectiveness in diagnostics (accuracy, speed, and timeliness), (2) impact on healthcare access and efficiency (reach in rural areas and resource optimization), and (3) barriers and facilitators of implementation (infrastructure, ethics, regulation, training, and cultural acceptance). Where possible, effect sizes, odds ratios, and confidence intervals were reported to describe quantitative findings. Narrative summary and the incorporation of the qualitative results into a topical compass were performed.

Results

The search of online databases yielded 380 studies. Following the removal of 84 duplicates, 296 titles and abstracts were screened, leading to the exclusion of 202 articles, as they did not meet the inclusion criteria. Out of those 94 full-text articles, 68 have been excluded due to irrelevance in focus, inappropriate study design, or being non-peer-reviewed materials such as editorials or commentaries. Ultimately, 26 studies were incorporated in the systematic review.

Geographically, 10 (38.5%) studies were in North America, six (23.1%) in Asia, five (19.2%) in Europe, and five (19.2%) in multiregional/low- and middle-income countries (LMICs). Included studies were 12 observational studies, seven implementation studies or case studies, and seven systematic reviews including both qualitative and quantitative evidence.

Thematic analysis identified three key areas: (1) access, in which the use of AI tools improved healthcare access in rural areas through telehealth, digitized decision support, and mobile applications modified diagnostic services; (2) efficiency and diagnostic accurateness, as AI technologies showed a speedier and more precise set of diagnoses; and (3) implementation barriers and enablers, such as infrastructural deficiencies, provider readiness, ethical issues, and community-level acceptability. In general, AI instruments can be effective in reducing healthcare disparities among underserved populations when obstacles within the system are overcome.

Table 2 provides a summary of the studies included in this systematic review, detailing study characteristics including study design, study population, and key findings.

**Table 2:** Summary of the included studies AI, artificial intelligence; LMICs, low- and middle-income countries; CHWs, community health workers; TB, tuberculosis; ML, machine learning; DL, deep learning

Reference	Study Design	Study Population	Key Findings
Gizaw et al. (2022) [1]	Systematic review	Rural communities	Improved infrastructure, workforce incentives, and community participation enhance primary healthcare access
Nelson et al. (2025) [2]	Qualitative systematic review	Community-based diagnostics implementers	Barriers include cost, training, and infrastructure; facilitators include community trust and integration
Weinhold and Gurtner (2014) [3]	Policy analysis review	Rural health systems	Workforce shortages and funding gaps explain persistent rural healthcare inequities
Getnet et al. (2017) [4]	Systematic review and meta-analysis	Patients with tuberculosis from LMIC	Significant diagnostic delays due to patient, provider, and system-level barriers
Golembiewski et al. (2022) [5]	Systematic review and synthesis	Rural patients with chronic conditions	Challenges include transport, cost, and fragmented care; need for patient-centered models
Tsou et al. (2021) [6]	Systematic review	Rural/remote emergency departments	Telehealth improved timeliness and access; implementation barriers include connectivity and staffing
Kumar et al. (2023) [7]	Systematic review	General healthcare	AI increasingly applied in diagnosis; gaps in integration and interpretability remain
Younis et al. (2024) [8]	Systematic review and meta-analysis	Medical AI applications	AI shows diagnostic promise; limitations include bias, regulation, and infrastructure
Hansun et al. (2025) [9]	Systematic review	TB diagnostic AI methods	AI-based imaging achieves high sensitivity/specificity but requires local validation
Saif-Ur-Rahman et al. (2023) [10]	Systematic review	LMIC primary care	AI/digital health tools improve efficiency but are hindered by infrastructure and governance gaps
Perez et al. (2025) [11]	Systematic review	Rural communities	AI and telemedicine enhance access but require sustained investment and trust-building
Abbasgholizadeh et al. (2021) [12]	Systematic scoping review	Community-based primary care	AI supports decision-making but has limited evidence of long-term outcomes
Ahmed et al. (2023) [13]	Systematic review	General healthcare	Barriers include cost, a lack of trust, regulation, and workforce readiness
Tang et al. (2023) [14]	Systematic review	Healthcare AI studies	Ethical concerns include bias, transparency, and patient autonomy
Ciecierski-Holmes et al. (2022) [15]	Scoping review	LMIC health systems	AI may strengthen systems but faces data, equity, and ethical challenges
Shahmoradi et al. (2017) [18]	Systematic review	Healthcare organizations	Knowledge management tools support evidence-based AI adoption
Dzabeng et al. (2016) [19]	Systematic review protocol	CHWs in LMICs	Explores experiences with mobile decision support for maternal/child health
Kaushik et al. (2025) [20]	Systematic review + case study	LMIC AI developers	Data sharing limited by privacy, infrastructure, and governance issues
Mennella et al. (2024) [21]	Review	Healthcare AI ethics	AI raises regulatory, ethical, and accountability challenges
Siala and Wang (2022) [22]	Systematic review	Healthcare AI studies	Responsible AI requires fairness, inclusivity, and transparency
Mohammed et al. (2025) [23]	Systematic review	Nursing/clinical AI	Ethical and regulatory considerations critical for AI in nursing practice
Alam et al. (2024) [24]	Scoping review	Bangladesh healthcare	Big data, AI, and ML/DL can transform care but are limited by resources
Kim et al. (2024) [25]	Scoping review	Low-resource ultrasound	AI for point-of-care ultrasound improves diagnostics but faces implementation barriers
Guo and Li (2018) [26]	Review	Rural developing countries	AI can address shortages but requires policy and infrastructure support
d’Elia et al. (2022) [27]	Scoping review	Primary care	AI risks widening inequities unless equity-focused frameworks are applied
Ayorinde et al. (2024) [28]	Systematic review	Healthcare professionals	Mixed experiences: efficiency gains versus distrust and workload concerns

Table 3 presents the quality assessment of the included studies using the R-AMSTAR tool.

**Table 3:** Systematic review of quality R-AMSTAR evaluation Item 1, a priori design; item 2, duplicate study selection and data extraction; item 3, comprehensive literature search; item 4, publication status as an inclusion criteria; item 5, list of included and excluded studies; item 6, characteristics of included studies; item 7, documented assessment of the scientific quality of included studies; item 8, appropriate use of the scientific quality in forming conclusions; item 9, appropriate use of methods to combine study findings; item 10, assessment of publication bias likelihood; item 11, conflict of interest documentation. Methodological quality was assessed using the R-AMSTAR tool [17] R-AMSTAR: Revised Assessment of Multiple Systematic Reviews

Study (Year)	1	2	3	4	5	6	7	8	9	10	11	Score
Gizaw et al. (2022) [1]	1	1	1	1	1	1	0	1	1	1	1	10
Nelson et al. (2025) [2]	1	1	1	1	1	0	1	1	1	1	1	10
Weinhold and Gurtner (2014) [3]	1	1	1	0	1	1	0	0	1	1	1	8
Getnet et al. (2017) [4]	1	1	1	1	1	1	0	1	1	1	0	9
Golembiewski et al. (2022) [5]	1	1	1	1	1	1	1	1	1	1	1	11
Tsou et al. (2021) [6]	1	1	1	1	1	1	0	1	1	1	1	10
Kumar et al. (2023) [7]	1	1	1	0	1	1	1	0	1	1	1	9
Younis et al. (2024) [8]	1	1	1	1	1	1	1	1	1	1	0	10
Hansun et al. (2025) [9]	1	1	1	1	1	1	0	1	1	1	1	10
Saif-Ur-Rahman et al. (2013) [10]	1	1	1	0	1	1	0	0	1	1	1	8
Perez et al. (2025) [11]	1	1	1	1	1	1	0	1	1	1	1	10
Abbasgholizadeh et al. (2021) [12]	1	1	1	0	1	1	0	0	1	1	1	8
Ahmed et al. (2023) [13]	1	1	1	1	1	1	1	1	1	1	0	10
Tang et al. (2023) [14]	1	1	1	0	1	1	0	1	1	1	1	9
Ciecierski-Holmes et al. (2022) [15]	1	1	1	1	1	1	0	1	1	1	1	10
Shahmoradi et al. (2017) [18]	1	1	1	0	1	1	1	0	1	1	1	9
Dzabeng et al. (2016) [19]	1	1	1	1	1	1	1	1	1	1	0	10
Kaushik et al. (2025) [20]	1	1	1	1	1	1	0	0	1	1	1	9
Mennella et al. (2024) [21]	1	1	1	0	1	1	0	1	1	1	1	9
Siala et al. (2022) [22]	1	1	1	1	1	1	0	1	1	1	1	10
Mohammed et al. (2025) [23]	1	1	1	0	1	1	1	0	1	1	1	9
Alam et al. (2024) [24]	1	1	1	1	1	1	0	1	1	1	1	10
Kim et al. (2024) [25]	1	1	1	1	1	1	0	0	1	1	1	9
Guo and Li (2018) [26]	1	1	1	1	1	1	1	1	1	1	0	10
d’Elia et al. (2022) [27]	1	1	1	0	1	1	0	0	1	1	1	8
Ayorinde et al. (2024) [28]	1	1	1	1	1	1	1	1	1	1	1	11

Study findings

This systematic review revealed that methods such as outreach services or mobile clinics may help to increase access to primary healthcare (PHC) service provision among rural groups. The highest percentage of the population of developing countries is in rural places where doctor services are inaccessible. Rural communities travel to major cities to get specialist services [1]. Although barriers and catalyzers may vary depending on location or the type of facility, many are common across both, for example, training of the testing devices or the ease of using the machine [2]. The geographic disparities in the availability of adequate healthcare in the rural region were condensed into five categories: provider shortages, maldistribution, quality deficiencies, access limitation, and the inadequate utilization of healthcare services [3].

Patients in rural areas in Ethiopia have the health post (the first level of the health system) as most of their only source of primary access to care, and there is no TB etiologic service in the health post, and patients may have to travel a number of hours before reaching hospitals or health centers [4]. Healthcare is an ancient early adaptor when it comes to overall innovative technologies. Artificial intelligence and its sub-branches, machine and deep learning, today are headed to becoming the primary means of the healthcare system, including the generation of new health check activities to process patient accounts and records [7].

In the present-day healthcare, the magnitude of information and knowledge processing demand is so immense due to massive data and information collection that every healthcare service provider collects each day [19]. As training and modifications continue, it is projected that the generalizability of ultrasound AI models will become better. Many of the articles reviewed were based on pilot studies [26].

However, in several studies, healthcare professionals indicated that AI tools offered value addition and better decision-making. These involved the elimination of tedious chores, the prevention of errors, less variability in clinical practice, enhanced associations between teams, improved data gathering, and the performance of the teams [28].

The implementation of AI and telehealth in rural areas may not be as successful as most people think due to a variety of issues, such as poor access to broadband, digital literacy barriers, and data privacy concerns [11]. A significant workload was mentioned as one of the most common obstacles to medical AI-related technology application, so the automatic and smart way to overcome this is to employ an intelligent workload-reduction mechanism, such as voice recognition technology [26].

Nevertheless, most current AI-based studies cannot be readily adopted by most AI because they do not follow a unified approach to AI governance, one that is ethical, regulatory, and practical [20]. Even though AI ethical frameworks have been revised several times to capture the complexity of AI ethical challenges, they offer less information regarding what efforts ought to be pursued to promote responsible AI use [22].

Discussion

Barriers and Facilitators to Access in Rural Healthcare

Telemedicine may be an effective substitute to the healthcare system that exists when there are common medical issues identified, questions concerning different medical concerns on home treatments, follow-ups or check-ins during post-treatment, or chronic care. Technology costs may also comprise an obstacle [1]. Among the leading contributors to the inability of Ghana and most developing nations to achieve their health-related Millennium Development Goals (MDGs) are poor literacy levels, cultural issues, poor quality care at the community level, and poor service demand by women [19].

Other studies indicate that rural accessibility barriers of developing countries can be overcome by using low-cost diagnostic tools that substitute more costly or challenging traditional screening devices found in traditional places inaccessible in rural locations [26].

Applications and Effectiveness of AI-Driven Diagnostic Tools

Although the technical possibilities of AI in the various fields of medicine, such as ophthalmology and radiology, had improved significantly, in many studies, which could not have been included in the review, there were proofs of concepts, which failed to describe the AI implementations in the real, low-resource context, preventing us to get a proper idea of the actual performance of AI and their advantages therein [15]. Even though AI in LMICs may solve health disparities, LMICs have technical, political, legal, policy, and organizational limitations to data sharing, thus hindering adequate AI development and adoption. When tested in a local context, most of these barriers were relevant [19]. Another study explains that among the 77 studies, the majority (n = 65, 84%) used ML models. Fewer studies have used AI (4/77, 5%), DL (7/77, 9%), and big data analytics (BDA) (1/77, 1%) [24].

How AI has been implemented is part and parcel with how it works best with the systems in place and the general society and, by extension, how it influences human intelligence (HI). Several articles addressed the threat of having AI-augmented interventions target healthy, well-to-do, young people. The reason is that the disruptive nature of AI allows commercial providers to go beyond reliance on relatively expensive and complex human clinicians [27].

The integration of telehealth-enabled AI diagnostics into rural healthcare systems can significantly enhance healthcare accessibility, despite implementation and equity challenges. In rural emergencies, telehealth AI diagnostic tools have been considered effective when their use leads to improved or equivalent clinical or service use outcomes. For example, a notable reduction in local hospital length of stay was seen as favorable because of the promptness of radiologic evaluation facilitated through telehealth [6].

Beyond emergency situations, telehealth AI diagnostics can broaden access to specialist diagnostics and facilitate timely communication between patients in remote areas and healthcare providers, reducing both travel expenses and lengthy commutes [11]. However, the lack of reliable high-speed internet in many rural communities challenges the dependability of these systems, since AI diagnostics often depend on cloud-based platforms for real-time data sharing. Furthermore, rural healthcare providers have voiced concerns about not fully understanding AI-generated outputs or the reasoning behind them, which can undermine trust and hinder adoption [28].

Most of the studies found that healthcare professionals were worried that they would not comprehend the outputs of the AI or lack knowledge on why the outputs would be constructed in that manner. The issue was the confidence in the accuracy of the AI applications and recommendations. They found out that some healthcare professionals perceived AI as increasing value and improving decision-making, and others described it as the confirmation of their clinical thinking, and still, others found it of no use at all [28].

Ethical, Regulatory, and Implementation Considerations for AI in Rural and LMIC Healthcare

Furthermore, the European Commission has developed a new initiative: the Artificial Intelligence Act (AIA), which is meant to tackle various types of risks associated with the widespread adoption of AI [21]. This body of rules promotes the safe use of AI and tries to prevent or mitigate possible harms caused by the implementation of particular technologies [21].

Scholars have proposed a number of solutions to reduce the effects of this kind of bias [22]. In clinical practice, the prediction task should be generalized, and various predictive models should be constructed by including the contextual variables to provide the least effect of an algorithm bias on clinical decisions. Furthermore, the performance of AI models and algorithms should be checked regularly by algorithmic stewardship programs so that it becomes acceptable [22]. The other study claims that AI technologies bring significant opportunities to the field of nursing, especially in decision-making and task efficiency. Nevertheless, these benefits should be instilled by ethical considerations, such as safeguarding the rights of patients, the transparency of algorithms, and bias reduction. Existing regulatory provisions should be changed to address the ethical demands of nursing [23].

Limitations

This review has several limitations that should be acknowledged. First, only studies published in the English language between January 2010 and April 2025 were included, which may have omitted relevant research published in other languages or earlier foundational work. Second, conference abstracts, dissertations, and grey literature were excluded, potentially limiting the capture of emerging or pilot evidence in this rapidly evolving field. Third, as this review synthesizes review-level evidence, findings are dependent on secondary data extraction and may be subject to reporting biases and methodological limitations inherent in the included reviews.

Additionally, the availability of primary data remains limited in many rural and low-resource settings, where awareness, infrastructure, and adoption of AI technologies may be low, particularly in developing regions. This constrains the depth of analysis and underscores the need for more context-specific primary research. Finally, although key barriers and facilitators were identified, variability across health systems, policy environments, and resource contexts may limit the generalizability of the findings.

## Conclusions

The systematic review shows that diagnostic tools using AI can help increase access to and the efficiency of healthcare in underserved regions and rural areas due to their ability to facilitate prompt diagnoses, leverage scarce resources, and strengthen the capabilities of frontline providers. However, these technologies have been shown to help decrease inequities when the infrastructural, ethical, and regulatory obstacles are properly resolved. Nevertheless, implementation is still hampered by a lack of digital infrastructure, provider preparedness, and data privacy and trust issues. To policymakers, healthcare professionals, and technology builders alike, the findings also reinstated the essentiality of contextually sensitive approaches, long-term investment, and ethical ownership to ensure that AI solutions will realistically work toward enhanced, equitable healthcare delivery.
